# Comparison of cooperative learning through use of an immersive virtual reality anatomy model and a 3D plastic anatomical model

**DOI:** 10.1186/s12909-025-07397-z

**Published:** 2025-05-30

**Authors:** Shin-Yun Chen, Daniel Salcedo, Bu-Yuan Hsiao, Wen-Cheng Huang, Bor-Chyuan Su, Jiun-Lin Horng

**Affiliations:** 1 Department of Anatomy and Cell Biology, School of Medicine, College of Medicine, Taipei Medical University, Taipei, 11031 Taiwan; 2 Center for Interprofessional Engagement and Simulation, Belmont University, Nashville, TN USA; 3 Division of Cardiology and Cardiovascular Research Center, Department of Internal Medicine, Taipei Medical University Hospital, Taipei, 11031 Taiwan; 4 Division of Cardiology, Department of Internal Medicine, School of Medicine, College of Medicine, Taipei Medical University, Taipei, 11031 Taiwan; 5 Taipei Heart Institute, Taipei Medical University, Taipei, 11031 Taiwan; 6 Department of Emergency, School of Medicine, College of Medicine, Taipei Medical University, Taipei, 11031 Taiwan; 7 Emergency Department, Department of Emergency and Critical Care Medicine, Wan Fang Hospital, Taipei Medical University, Taipei, 11696 Taiwan; 8 Center for Education in Medical Simulation, Taipei Medical University, Taipei, 11031 Taiwan; 9 Department of Education and Humanities in Medicine, School of Medicine, College of Medicine, Taipei Medical University, Taipei, 11031 Taiwan

**Keywords:** Plastic anatomical model, Cooperative learning, Virtual reality, Anatomical structure

## Abstract

**Background:**

Traditional anatomy education relies on lectures, visual aids, and cadaver dissections. However, limited cadaver availability often necessitates the use of plastic models to aid 3D understanding. Virtual reality (VR) presents an immersive alternative that may enhance spatial learning without requiring cadavers. Despite its potential, few studies have directly compared VR with traditional methods in anatomy education.

**Objective:**

This study aimed to compare the learning outcomes of first-year anatomy students using either VR or plastic 3D models for anatomical instruction.

**Methods:**

First-year anatomy students were divided into two groups: one using VR and the other using plastic models. They participated in weekly anatomy sessions consisting of 2-hour lectures followed by 2-hour laboratory sessions covering various anatomical systems. After the lectures, students engaged in laboratory activities using either plastic models or immersive virtual reality (iVR) for 3D spatial anatomy learning, with iVR participants capturing screenshots of assigned targets for verification. Each session concluded with an online image-based multiple-choice quiz to assess anatomical identification and understanding.

**Results:**

Students from the Department of Nutrition and Health Sciences (NHS) and the Department of Medical Laboratory Science and Biotechnology (MLSB) at Taipei Medical University (TMU) participated in the study. Students in the VR group initially struggled due to the time required to adapt to the system, which was reflected in their significantly lower scores in week 2 for both NHS (80.35 ± 2.04 vs. 88.82 ± 1.64, *p* < 0.0019) and MLSB (72.23 ± 1.81 vs. 88.55 ± 1.67, *p* < 0.0001). However, in subsequent weeks, while iVR scores were slightly lower, the differences were not statistically significant, and by the later weeks, there was no significant difference in quiz performance between the two groups, with comparable scores observed in weeks 8 and 10 for NHS.

**Conclusions:**

VR provides a viable alternative to plastic models for anatomy education. Although students require an adaptation period, their performance eventually matches that of students using traditional plastic models. This study is the first to quantitatively compare VR and plastic models in anatomy instruction.

**Supplementary Information:**

The online version contains supplementary material available at 10.1186/s12909-025-07397-z.

## Introduction

Traditional methods of teaching gross anatomy in medical schools typically involve a combination of classroom lectures supported by visual aids such as slides, figures, or medical imaging, along with hands-on dissection laboratories where students either perform dissections themselves or observe pre-dissected specimens and anatomical models [1, 2]. Due to the limited time allocated for cadaver observation during scheduled class hours, students often rely heavily on textbooks, lecture recordings, and two-dimensional (2D) anatomical atlases for self-study [3, 4].

Understanding the human body in three dimensions (3D) is crucial for mastering anatomical structures and spatial relationships. Effective learning in this field necessitates extensive training in 3D spatial thinking and visuospatial abilities [5, 6]. Direct dissection of cadavers provides the most comprehensive educational experience by allowing students to feel the texture of fascia and organs, and visually appreciate the depth and complexity of anatomical structures, thus enhancing long-term retention of anatomical knowledge [7, 8]. However, ethical and cultural constraints, particularly in countries like Taiwan where filial piety and respect for the deceased limit the availability of donated bodies, pose significant challenges to providing ample cadaveric material for many anatomy students [9]. As a result, medical schools often resort to using realistic plastic models, preserved prosection slides, and partial real organ displays as teaching aids in paramedical courses [10–12]. While such models are advantageous in building 3D spatial understanding and reducing cognitive load, their high cost poses a barrier to widespread implementation [13].

In recent years, the COVID-19 pandemic accelerated the adoption of digital learning technologies. Advancements in augmented reality, mixed reality (MR), and virtual reality (VR), combined with the use of head-mounted displays (HMDs), have paved the way for innovative learning resources [12, 14]. These technologies are invaluable for autonomous learning, as data indicate widespread acceptance of 3 C (computer, communication, and consumer electronics) products for educational purposes [15, 16].

VR learning environments created through extended reality technologies not only offer immersive learning experiences and realistic models but also provide high accessibility and convenience [17, 18]. Compared to traditional cadaver dissection, VR reduces the burden of body donation campaigns and regulatory constraints, while being more readily accepted by students familiar with digital technologies [19]. The literature suggests significant differences in learning outcomes between students who use VR and those who follow traditional methods of learning anatomy [20, 21]. In the context of anatomy education, VR can simulate the segmentation and reconstruction of anatomical structures, helping medical students develop their 3D imaging concepts, visuospatial skills, and anatomical localization [22, 23]. However, there is a scarcity of comprehensive studies comparing the learning efficacy of VR to traditional plastic models.

Educators have mixed opinions on the broad application of VR in teaching. While gamification, constructivist pedagogies, and group dynamics in VR are expected to enhance learning motivation, the high cost of VR programs designed for multiple users remains a concern [24, 25]. Additionally, prolonged use of VR equipment may cause a certain degree of harm, including eye strain and potential long-term ocular health issues [24]. Nonetheless, with appropriate guidance and moderation, MR technologies can serve as valuable tools for fostering independent, post-class learning among students [26].

In this article, we aimed to explore differences between VR and traditional plastic models in students’ learning of anatomical structures. We divided students into groups of five or six and assigned them approximately 30 anatomical terms per week to learn cooperatively using either VR or plastic 3D models. After 1–1.5 h of learning, a quiz was administered to measure learning effectiveness by matching anatomical structures with specialized terms using diagrams. Results showed that VR was less effective at the beginning of the course, as students reported needing time to learn how to use the VR system, which reduced their focus on anatomical structures. However, by the third week, the learning effectiveness test showed no significant difference between the VR and plastic 3D model groups on the quiz. Ours is the first study to quantify differences between VR and plastic 3D models on students’ anatomy learning outcomes in a quiz.

## Materials and methods

### Participants and course design

First-year students from the Department of Nutrition and Health Sciences (NHS) and Department of Medical Laboratory Science and Biotechnology (MLSB) of Taipei Medical University (TMU) participated, with the research approved by the TMU Joint Institute Review Board (TMU-JIRB; Approval No. N202104008). This study was conducted in accordance with the principles of the Declaration of Helsinki. Given that this research involved routine educational activities without interventions, we applied for a waiver of written informed consent, which was approved through an expedited review by the TMU-JIRB. However, students were orally informed about the study procedures before participation. The anatomy courses for NHS and MLSB are designed to include 2 h of lecture and 2 h of laboratory work per week. Each week focused on a specific anatomical system, including skeletal (2 weeks), muscular (2 weeks), cardiovascular (2 weeks), nervous (2 weeks), digestive (2 weeks), respiratory (1 week), urinary (1 week), male (1 week), and female (1 week) systems. Following a 2-hour traditional lecture session, students attended their laboratory courses to respectively use plastic models or immersive VR (iVR) to practice 3D spatial anatomy systems. Students from each department with similar university entrance exam scores consented to data collection.

### Hardware and software

The hardware used in the laboratory class was a desktop computer equipped with an Intel(R) Core(TM) i7-8700 CPU, 16.0 GB of DDR4 RAM, and an NVIDIA GeForce GTX 1080 Ti GPU, and HTC VIVE HMDs (with the VR environment rendered at a resolution of 1440 × 1600 pixels per eye at 90 Hz). The computer was equipped with Microsoft Office and Medicalholodeck AI (VR anatomy software). Participants engaged with the Medicalholodeck AI application via the HMDs and tactile controllers. As a student operator navigated the software, their view was simultaneously displayed on a computer screen, allowing other group members to assist in identifying target structures.

### Course preparation and grouping

Before engaging with the iVR in laboratory classes, students received a 15-minute English online tutorial, and a teaching assistant was present to assist with any operational issues during the class.

**Fig. 1:**
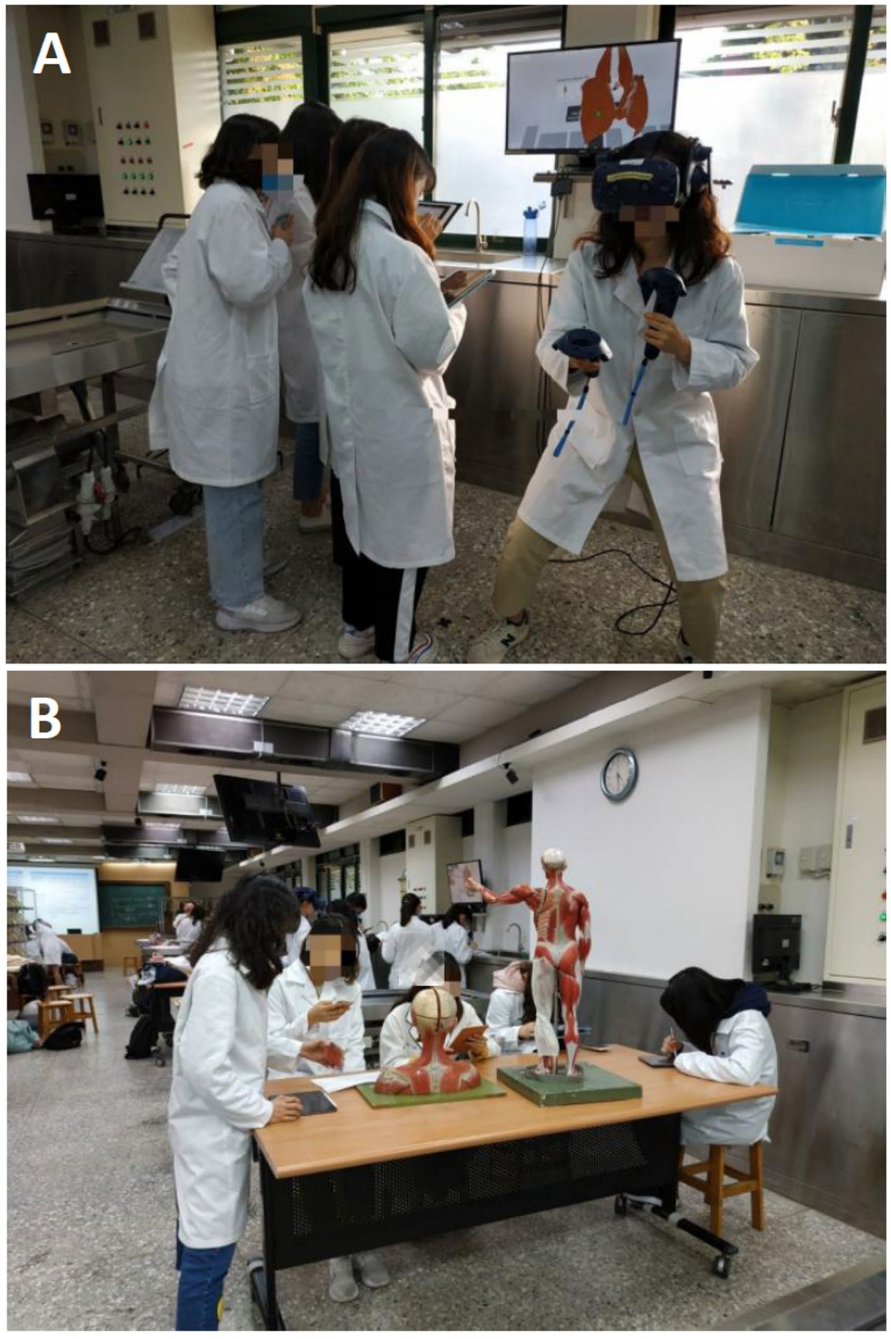
Schematic representation of students used immersive virtual reality (**A**) and a 3D plastic anatomical model (**B**)

**Fig. 2:**
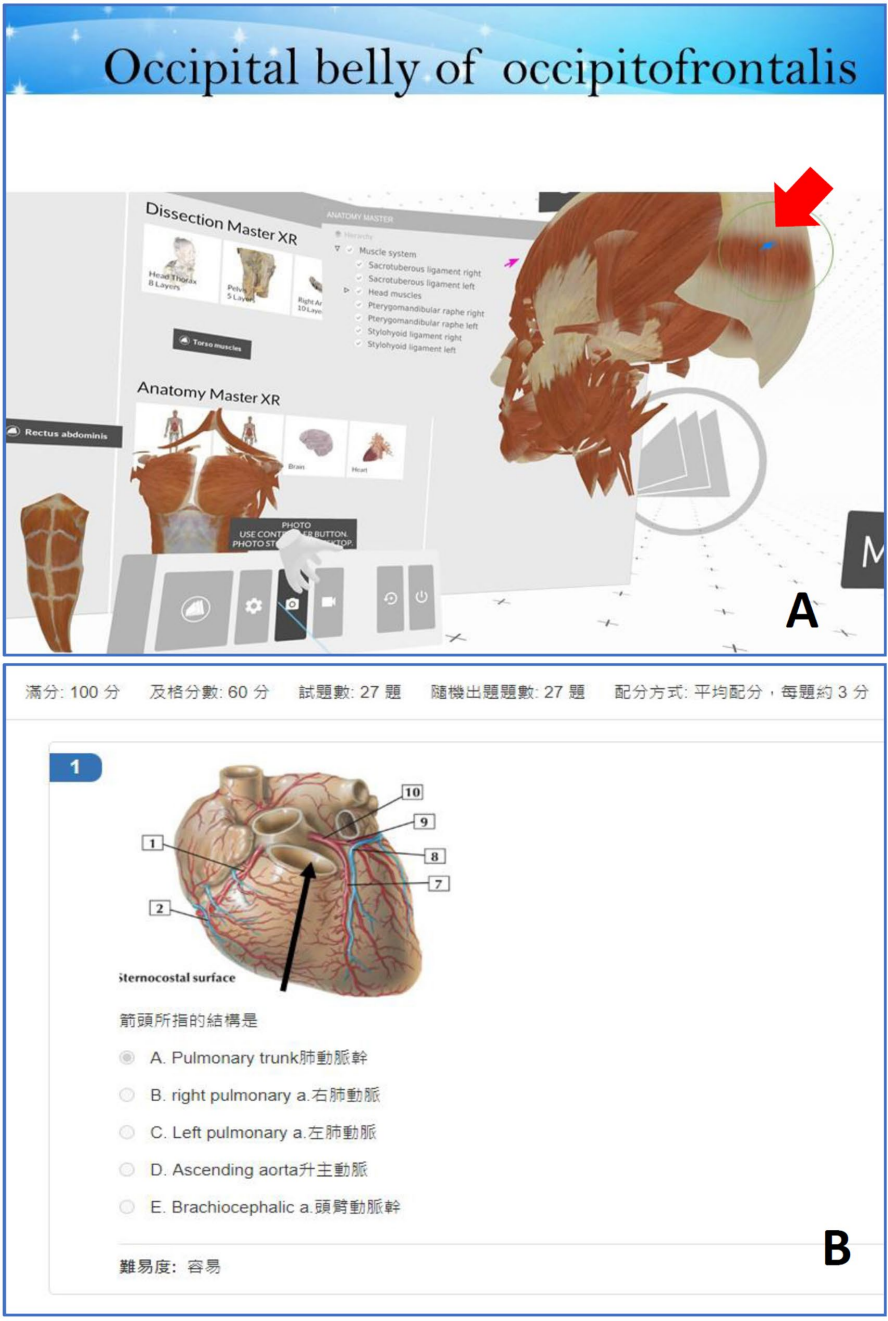
Class assignment and quiz. The immersive virtual reality (iVR) group had an additional assignment to take screenshots of the designated anatomical structure terms to verify their findings (**A**). Students had to mark the target (arrow in A). (**B**) The quiz was an image-based examination for which students had to identify the structure shown in the image by selecting the correct name from five options available in both English and Chinese

The anatomy laboratory course of NHS is a required course which included 69 students, divided into seven groups, each of which was further split into subgroups A and B (with ca. five students per group and with a similar ratio of males to females). The anatomy laboratory course of MLSB is an elective course that included 74 students, divided into six groups, each of which was further split into subgroups A and B (with ca. six students per group and with a similar ratio of males to females). At the beginning of the NHS laboratory course, ~ 30 anatomical terms were provided to each group as learning targets (table S1). Subgroup A observed plastic anatomical models, while subgroup B used the iVR software to identify and observe the 3D structure of anatomical terms (Fig. 1). The iVR group needed to complete an additional task using the built-in photo mode of the iVR system to take screenshots of the designated targets (anatomical structures) to verify their findings (Fig. 2A). All activities were completed within 60 min, after which students were assigned a quiz. In the second hour, a different list was given, and subgroups A and B were exchanged (data were not used). The class design of the MLSB anatomy laboratory course was the same as that of NHS. The only difference was that class activities were designed to be completed within 90 min, and subgroups A and B exchanged tasks every other week. During the course, every subgroup could practice with plastic anatomical models and iVR.

### Quizzes

The quiz was an image-based examination conducted online, using a multiple-choice format where students had to identify the structure shown in the image by selecting the correct name from five options, available in both English and Chinese (Fig. 2B). The examination questions were derived from Netter’s Anatomy Flash Cards by John T. Hansen, a widely recognized reference in anatomical education. The raw data have been de-identified and are available in the supplementary materials (table S2 and S3).

### Statistical analysis

Data are expressed as the mean ± standard error of the mean (SEM). The analysis was performed using GraphPad Prism vers. 9.0. The Shapiro-Wilk normality test was applied to evaluate the normality of the data before statistical analyses were performed. Student’s unpaired *t*-test was used to compare the means of the two groups. Statistical significance was accepted at *p* < 0.05.

## Results

### Group-based anatomy laboratory course

Students in the iVR group took turns using the iVR system, with each student using it for about 10–15 min, during which time no one expressed any discomfort. The iVR group had to perform an additional assignment to ensure that the anatomical term listed by the teacher was observed in the iVR software (Fig. 2A). Overall, 95% of the iVR assignments reviewed by the teacher showed complete structural observation, while 5% were incomplete due to omissions from the list.

### Comparison of scores between the plastic anatomical model and iVR groups in NHS

**Fig. 3:**
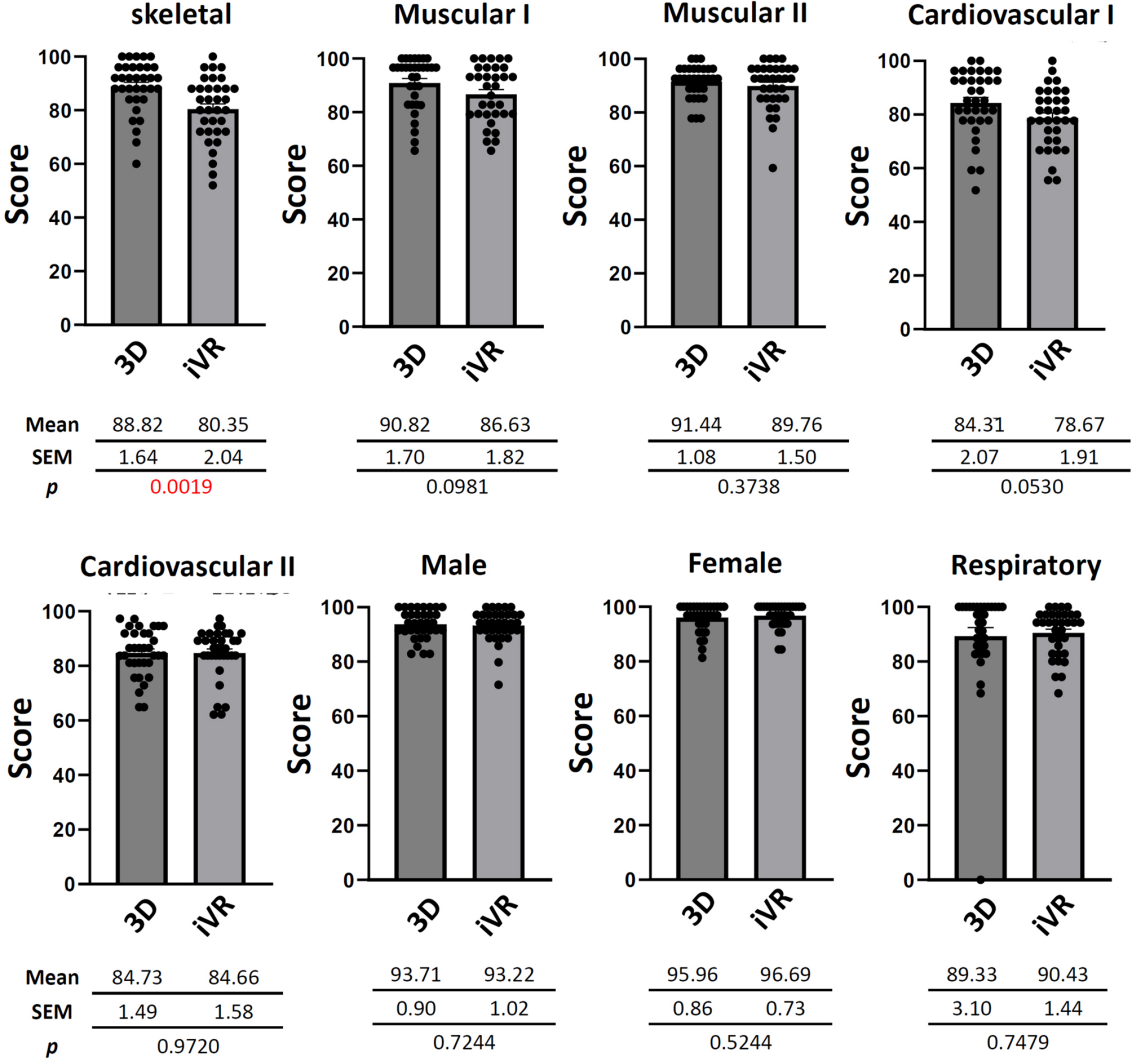
Bar chart representing a comparison of mean scores between the 3D plastic anatomical model group (3D) and immersive virtual reality group (iVR) on each quiz including the standard error of the mean (SEM) and *p* value, in Nutrition and Health Science

In weeks 2, 3, 4, 5, 6, 8, 9, and 10 of the anatomical laboratory course of NHS (respectively covering the skeletal, muscular I, muscular II, cardiovascular I, cardiovascular II, male, female, and respiratory systems), comparisons between the plastic anatomical model and iVR groups were conducted. A significant difference in scores was observed only in week 2 (skeletal system), where the iVR group scored significantly lower (80.35 ± 2.04) compared to the plastic anatomical model group (88.82 ± 1.64) (*p* < 0.0019; Fig. 3). With exceptions of weeks 8 and 10 (female and respiratory systems), scores of the iVR group in weeks 3, 4, 5, 6, and 9 were slightly lower than those of the plastic anatomical model group, but the differences were not statistically significant (Fig. 3).

### Comparison of scores between the plastic anatomical model and iVR groups in MLSB

**Fig. 4:**
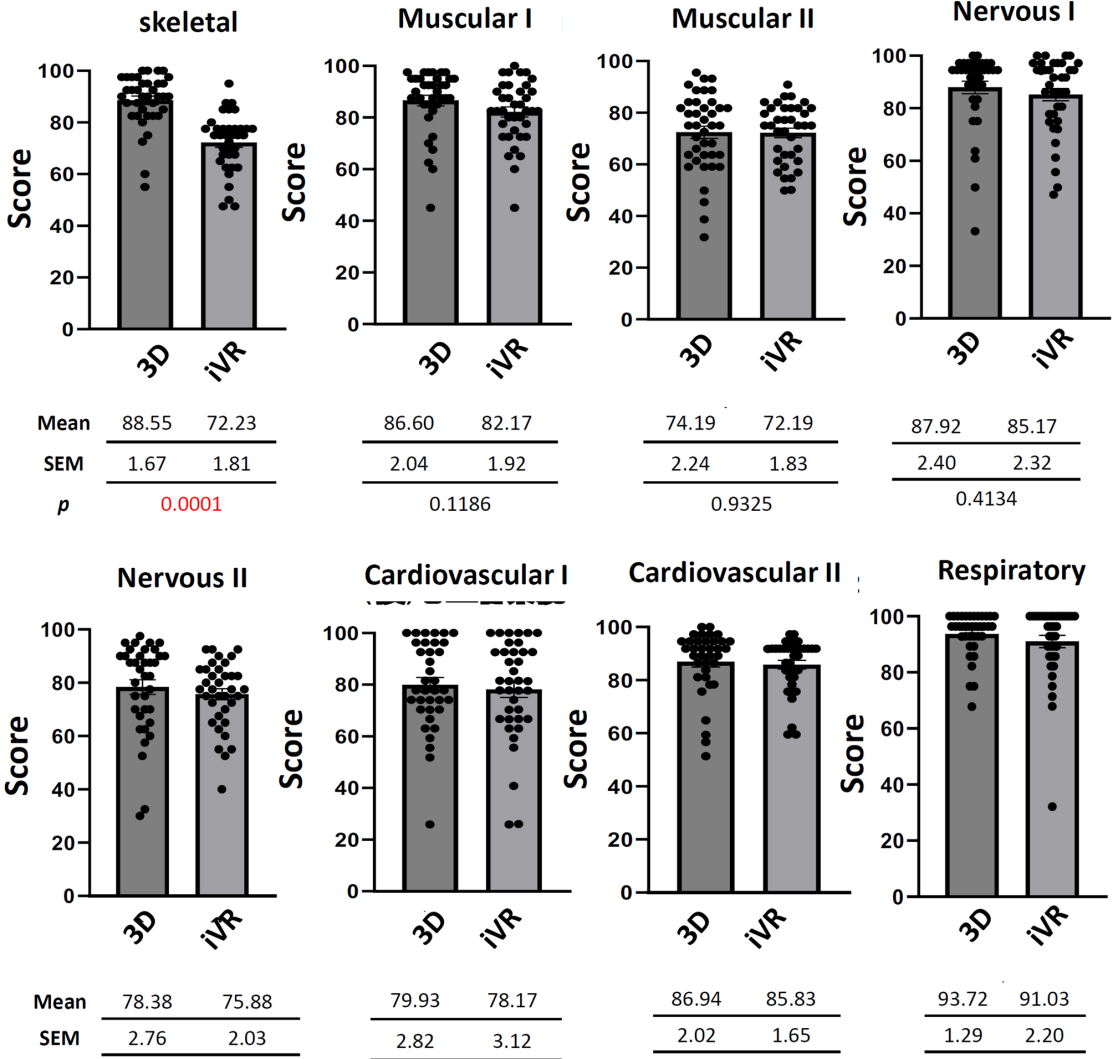
Bar chart representing a comparison of mean scores between the 3D plastic anatomical model group (3D) and immesrsive virtual reality group (iVR) on each quiz including the standard error of the mean (SEM) and *p* value, in Medical Laboratory Science and Technology

In weeks 2, 3, 4, 5, 6, 9, 10, and 11 of the anatomical laboratory course of MLSB (respectively covering skeletal, muscular I, muscular II, nervous I, nervous II, cardiovascular I, cardiovascular II, and respiratory systems) comparisons between the plastic anatomical model and iVR groups were conducted. Similarly, a significant difference in scores was observed only in week 2 (skeletal system), where the iVR group scored significantly lower (72.23 ± 1.81) compared to the plastic anatomical model group (88.55 ± 1.67) (*p* < 0.0001; Fig. 4). Scores of the iVR group in all other weeks (3, 4, 5, 6, 9, 10, and 11) were lower than those of the plastic anatomical model group, but the differences were not statistically significant (Fig. 4).

## Discussion

Ours is the first study to examine the learning effectiveness of systematic anatomy by administering an immediate test at the end of each class. The study was conducted to evaluate the effectiveness of different learning tools on students’ learning of anatomy. Results of the implementation in both classes showed that the modeling group had better learning outcomes than the iVR group in comparisons of learning outcomes at the beginning of the course. However, no significant differences were detected after week 3. This may have been due to the fact that at the beginning of the program, students were not yet familiar with the software or the operation of the hardware. Our results are in agreement with previous authors that there are indeed many advantages and disadvantages of using iVR as a pedagogical tool for learning anatomy in a cadaver resource-poor environment [24, 27, 28].

### Comparisons of results between NHS and MLSB

In the anatomy curriculum, the skeletal system is the first system presented to students, and we found that there was a significant decrease in iVR scores in both NHS and MLSB when the skeletal system course was taught using 3D models versus iVR models. One explanation for this trend is that the iVR group was not familiar with the iVR interface at the beginning of the course. Although there was a demonstration before the course, there were still a lot of things that students had to overcome when they first began to operate the program on their own; for example, familiarizing themselves with the iVR software interface, adapting to the immersive experience of HMDs, and acclimating to the new structure of 3D visual spatial awareness. Once students were familiar with the operation mode of iVR and overcame difficulties with its use, they could achieve the same learning effect regardless of whether they used plastic models or iVR [29]. Our results showed that there was no significant difference in learning between groups A and B in later stages of the course.

### iVR advantages

As to recommendations on the use of iVR when students use it to learn systematic anatomy, we agree with the conclusions of Ail et al. [1]. The most practical way to use iVR is to combine it with traditional teaching methods. The reason for this argument is that iVR consumes more time in the early stages of learning, and students are not yet familiar with the anatomical structures they have just learned. If the time for a single session is insufficient, relying on lectures and iVR alone may result in some students being unable to fully comprehend and retain the material within the limited time.

In addition, in classroom teaching, verbal explanations, textbooks, 2D electronic notes, and actual touching of 3D plastic models are still indispensable and the main tools for transferring knowledge. These traditional teaching methods allow students to quickly master the basics within the limited classroom time, laying a solid foundation for subsequent iVR operations. Subsequently, using iVR for more interactive and collaborative learning, you could further randomly select a few systems to watch from a dead-angle, so that students can visualize the anatomy in the classroom and build a 3D structure. Then, through iVR, students can engage in more interactive and collaborative learning, allowing them to freely select and view certain systems from all angles [30]. This would help students visualize and conceptualize anatomical structures they have learned in class in a more 3D way. This would allow for deeper integration of the structures from different systems within the same region, enabling students to better understand complex spatial relationships among structures and achieve a stronger connection between systemic and regional anatomy [31]. Additionally, providing the iVR application after class would allow students to flexibly use their extra time for stress-free, repetitive practice.

In 2017, Moro and colleagues (2017) [29] used a human skull as a research focus and showed that studies using VR and 3D models resulted in no significant differences in students’ learning of the skull between the two methods. This result is consistent with our findings from week 3 onwards. After becoming familiar with the software in week 2, students were able to easily replicate the same learning approach and apply it to other systems, achieving the same learning outcomes as with 3D models [24]. Due to students’ interest and attraction to the new software, they quickly adapted to the spatial awareness it provided and could easily apply it later on. This had a significantly positive impact on enhancing learning interest. The sense of accomplishment from mastering new tools through collaboration fostered greater initiative and continuity in learning, and may have further stimulated their exploration and application of new knowledge, extending their long-term memory. Future research strategies could focus on whether after entering clinical practice, students show improved understanding or proficiency in tasks that require spatial comprehension tools in related fields.

### iVR disadvantages

Many studies comparing 3D interactive models and VR to 2D images and textbooks found that iVR has higher computer hardware requirements. High-resolution graphics often need powerful computers and specialized VR equipment. Additionally, subscribing annually to very detailed anatomical software involves extra costs, and subsequent maintenance of hardware requires further investment of time and money [32]. Considering these factors, limited funding or being located in more rural areas may restrict the use of iVR in some schools. Users not only need to learn how to operate the system, but learners unfamiliar with VR may require more time to adapt. Additionally, some individuals may experience health issues such as cybersickness or eye strain after using the system, which increases cognitive loads and makes it difficult to use the equipment for extended periods [27, 28]. Intermittent use may help alleviate these physical symptoms, but discontinuous learning could potentially reduce enthusiasm for learning.

Compared to hand-drawn (computer-generated) anatomical software, real cadaveric dissection offers a more-authentic learning experience and fosters a better understanding of the diversity of the human body. Actual interaction with and manipulation of various human structures allow individuals to become familiar with the hardness, depth, and tactile feedback of different anatomical features. However, cadavers are limited by traditional customs (requirements for cremation and burial), making preservation difficult, and prolonged use can lead to the loss of certain subtle structures. Therefore, if VR anatomical software can be combined with computed tomography (CT) or magnetic resonance imaging (MRI), it would better represent real human anatomy, accurately displaying 3D structures and reflecting their diversity. Although many software programs can currently synthesize these images, presenting the finer structures individually in space and clearly displaying their orientations would enhance the effectiveness of these techniques in anatomical and clinical education. Additionally, by increasing the number of samples from CT or MRI, it would be possible to more effectively reveal the variability of human structures. This would not only help students gain a deeper understanding of individual differences but would also prepare them to face a wider range of structural variations before entering clinical practice, enhancing their clinical judgment and procedural skills.

## Electronic supplementary material

Below is the link to the electronic supplementary material.

Supplementary Material 1

Supplementary Material 2

Supplementary Material 3

## Data Availability

The raw quiz data have been de-identified and are available in the supplementary materials (Tables S2 and S3).

## Abbreviations

HMDs: Head-mounted displays
MR: Mixed reality
VR: Virtual reality

## References

[1] Ail G, Freer F, Chan CS, Jones M, Broad J, Canale GP, et al. A comparison of virtual reality anatomy models to prosections in station-based anatomy teaching. Anat Sci Educ. 2024;17(4):763–9. 10.1002/ase.2419. PMID: 38584323. 38584323 10.1002/ase.2419

[2] Sugand K, Abrahams P, Khurana A. The anatomy of anatomy: a review for its modernization. Anat Sci Educ. 2010;3(2):83–93. PMID: 20205265. 10.1002/ase.139 10.1002/ase.139 20205265

[3] McBride JM, Drake RL. National survey on anatomical sciences in medical education. Anat Sci Educ. 2018;11(1):7–14. 10.1002/ase.1760. PMID: 29265741. 29265741 10.1002/ase.1760

[4] Turney BW. Anatomy in a modern medical curriculum. Ann R Coll Surg Engl. 2007;89(2):104–7. 10.1308/003588407X168244. PMID: 17346399. 17346399 10.1308/003588407X168244 PMC1964553

[5] Langlois J, Bellemare C, Toulouse J, Wells GA. Spatial abilities and anatomy knowledge assessment: a systematic review. Anat Sci Educ. 2017;10(3):235–41. 10.1002/ase.1655. PMID: 27731946. 27731946 10.1002/ase.1655

[6] Fernandez R, Dror IE, Smith C. Spatial abilities of expert clinical anatomists: comparison of abilities between novices, intermediates, and experts in anatomy. Anat Sci Educ. 2011;4(1):1–8. PMID: 21265030. 10.1002/ase.196 10.1002/ase.196 21265030

[7] Ghosh SK. Cadaveric dissection as an educational tool for anatomical sciences in the 21st century. Anat Sci Educ. 2017;10(3):286–99. 10.1002/ase.1649. PMID: 27574911. 27574911 10.1002/ase.1649

[8] Radzi S, Chandrasekaran R, Peh ZK, Rajalingam P, Yeong WY, Mogali SR. Students’ learning experiences of three-dimensional printed models and plastinated specimens: a qualitative analysis. BMC Med Educ. 2022;22(1):695. PMID: 36171608. 10.1186/s12909-022-03756-2 10.1186/s12909-022-03756-2 PMC9520930 36171608

[9] Winkelmann A. Consent and consensus-ethical perspectives on obtaining bodies for anatomical dissection. Clin Anat. 2016;29(1):70–7. 10.1002/ca.22651. PMID: 26475682. 26475682 10.1002/ca.22651

[10] Fruhstorfer BH, Palmer J, Brydges S, Abrahams PH. The use of plastinated prosections for teaching anatomy–the view of medical students on the value of this learning resource. Clin Anat. 2011;24(2):246–52. 10.1002/ca.21107. PMID: 21322047. 21322047 10.1002/ca.21107

[11] Goh JSK, Chandrasekaran R, Sirasanagandla SR, Acharyya S, Mogali SR. Efficacy of plastinated specimens in anatomy education: a systematic review and meta-analysis. Anat Sci Educ. 2024;17(4):712–21. 10.1002/ase.2424. PMID: 38591116. 38591116 10.1002/ase.2424

[12] Davis CR, Bates AS, Ellis H, Roberts AM. Human anatomy: let the students tell us how to teach. Anat Sci Educ. 2014;7(4):262–72. 10.1002/ase.1424. PMID: 24249485. 10.1002/ase.1424 24249485

[13] Rani S, Gupta GK, Chakraborty R, Kumar T, Kumar MK, Das AK, et al. Comparative analysis of multidimensional learning tools in anatomy: a randomized control trial. Ann Afr Med. 2024;23(3):459–65. 10.4103/aam.aam_214_23. PMID: 39034573. 39034573 10.4103/aam.aam_214_23 PMC11364337

[14] O’Neill S, Galbraith G, Enterline R, Wish-Baratz S. Student perceptions of superimposed mixed reality anatomy: a bridge between the virtual and physical. Med Sci Educ. 2023;33(2):343-4. PMID: 37261013. 10.1007/s40670-023-01763-6 10.1007/s40670-023-01763-6 PMC10226936 37261013

[15] Birbara NS, Sammut C, Pather N. Virtual reality in anatomy: a pilot study evaluating different delivery modalities. Anat Sci Educ. 2020;13(4):445–57. 10.1002/ase.1921. PMID: 31587471. 31587471 10.1002/ase.1921

[16] Turso-Finnich T, Jensen RO, Jensen LX, Konge L, Thinggaard E. Virtual reality head-mounted displays in medical education: a systematic review. Simul Healthc. 2023;18(1):42–50. 10.1097/SIH.0000000000000636. PMID: 35136005. 35136005 10.1097/SIH.0000000000000636

[17] Preece D, Williams SB, Lam R, Weller R. Let’s get physical: advantages of a physical model over 3D computer models and textbooks in learning imaging anatomy. Anat Sci Educ. 2013;6(4):216–24. 10.1002/ase.1345. PMID: 23349117. 10.1002/ase.1345 23349117

[18] Stepan K, Zeiger J, Hanchuk S, Del Signore A, Shrivastava R, Govindaraj S, et al. Immersive virtual reality as a teaching tool for neuroanatomy. Int Forum Allergy Rhinol. 2017;7(10):1006–13. 10.1002/alr.21986. PMID: 28719062. 28719062 10.1002/alr.21986

[19] Gloy K, Weyhe P, Nerenz E, Kaluschke M, Uslar V, Zachmann G et al. Immersive anatomy atlas: learning factual medical knowledge in a virtual reality environment. Anat Sci Educ. 2022;15(2):360-8. PMID: 33896115. 10.1002/ase.2095 10.1002/ase.2095 33896115

[20] Wang CY, Yin T, Ma KH, Shyu JF, Cheng CP, Wang YC et al. Enhancing anatomy education through cooperative learning: harnessing virtual reality for effective gross anatomy learning. J Microbiol Biol Educ. 2023;24(3). PMID: 38108010. 10.1128/jmbe.00100-23 10.1128/jmbe.00100-23 PMC10720469 38108010

[21] Trelease RB. From chalkboard, slides, and paper to e-learning: how computing technologies have transformed anatomical sciences education. Anat Sci Educ. 2016;9(6):583–602. 10.1002/ase.1620. PMID: 27163170. 27163170 10.1002/ase.1620

[22] Chauhan P, Mehra S, Pandya A. Randomised controlled trial: role of virtual interactive 3-dimensional models in anatomical and medical education. J Vis Commun Med. 2024;47(1):39–45. 10.1080/17453054.2024.2352404. PMID: 38767329. 38767329 10.1080/17453054.2024.2352404

[23] Wainman B, Pukas G, Wolak L, Mohanraj S, Lamb J, Norman GR. The critical role of stereopsis in virtual and mixed reality learning environments. Anat Sci Educ. 2020;13(3):401–12. 10.1002/ase.1928. PMID: 31665563. 31665563 10.1002/ase.1928

[24] Mergen M, Graf N, Meyerheim M. Reviewing the current state of virtual reality integration in medical education - a scoping review. BMC Med Educ. 2024;24(1):788. PMID: 39044186. 10.1186/s12909-024-05777-5 10.1186/s12909-024-05777-5 PMC11267750 39044186

[25] Liao ML, Yeh CC, Lue JH, Chang MF. Implementing virtual reality technology to teach medical college systemic anatomy: a pilot study. Anat Sci Educ. 2024;17(4):796–805. 10.1002/ase.2407. PMID: 38487974. 38487974 10.1002/ase.2407

[26] Birt J, Stromberga Z, Cowling M, Moro C. Mobile mixed reality for experiential learning and simulation in medical and health sciences education. Information. 2018;9(2):31. PMID: 10.3390/info9020031

[27] Jallad ST, Natsheh I, Helo LA, Ibdah DM, Salah A, Muhsen R et al. Nursing student’s perceptions, satisfaction, and knowledge toward utilizing immersive virtual reality application in human anatomy course: quasi-experimental. BMC Nurs. 2024;23(1):601. PMID: 39198772. 10.1186/s12912-024-02254-8 10.1186/s12912-024-02254-8 PMC11361164 39198772

[28] Adnan S, Michael P, Benson AC, Xiao J. Junior and senior students possess differential preferences towards multimodal digital anatomy resources. Clin Anat. 2024. PMID: 38716865. 10.1002/ca.24175 10.1002/ca.24175 38716865

[29] Moro C, Štromberga Z, Raikos A, Stirling A. The effectiveness of virtual and augmented reality in health sciences and medical anatomy. Anat Sci Educ. 2017;10(6):549–59. 28419750 10.1002/ase.1696

[30] Fairen M, Moyes J, Insa E. VR4Health: personalized teaching and learning anatomy using VR. J Med Syst. 2020;44(5):94. 10.1007/s10916-020-01550-5. PMID: 32193612. 32193612 10.1007/s10916-020-01550-5 PMC7082407

[31] Deng X, Zhou G, Xiao B, Zhao Z, He Y, Chen C. Effectiveness evaluation of digital virtual simulation application in teaching of gross anatomy. Ann Anat. 2018;218:276–82. 10.1016/j.aanat.2018.02.014. PMID: 29679721. 29679721 10.1016/j.aanat.2018.02.014

[32] Jiang H, Vimalesvaran S, Wang JK, Lim KB, Mogali SR, Car LT. Virtual reality in medical students’ education: scoping review. JMIR Med Educ. 2022;8(1):e34860. 10.2196/34860. PMID: 35107421. 35107421 10.2196/34860 PMC8851326

