# Supplementary material for: Comparison of cooperative learning through use of an immersive virtual reality anatomy model and a 3D plastic anatomical model
Source: BMC Med Educ. 2025 May 30;25:807. doi: 10.1186/s12909-025-07397-z (PMC12125823; doi:10.1186/s12909-025-07397-z)
Supplement: Supplementary file 1 — Supplementary Material 1 [file 12909_2025_7397_MOESM1_ESM.docx]

| Table S1 Anatomical term checklist every week | | |
| --- | --- | --- |
| Department of Nutrition and Health Sciences | | |
| Week | Topic | checklist |
| 2 | Skeletal_check list (20) | Coronoid process, Styloid process of ulna, Styloid process of radius, Acetabulum, Iliac crest, Iliac fossa, Ischial spine, Ischial tuberosity, Obturator foramen, Pubic symphysis, Greater sciatic notch, Greater trochanter, Lesser trochanter, Neck of femur, Patellar surface, Patella, Tibial tuberosity, Medial malleolus, Lateral malleolus, Head of fibula |
| 3 | Muscular I_check list (20) | frontal belly of occipitofrontalis, Occipital belly of occipitofrontalis, Orbicularis oculi, Zygomaticus major, Zygomaticus minor, Risorius, Levator labii superioris, Depressor labii inferioris, Depressor anguli oris, mentalis, Orbicularis oris, Buccinator, Platysma, Auricular, Masseter, Temporalis, sternal head of sternocleidomastoid, clavicular head of sternocleidomastoid, Medial pterygoid, Lateral pterygoid |
| 4 | Muscular II_check list (20) | flexor digitorum superficialis, flexor pollicis longus, extensor carpi ulnaris, abductor pollicis longus, brachioradialis, Sartorius, adductor longus, Iliopsoas, adductor magus, Quadriceps femoris, Gluteus maximus, Gluteus medius, biceps femoris, semitendinosus, semimembranosus, Gastrocnemius, Soleus, Flexor digitorum longus, Flexor hallucis, longus, Tibialis posterior, |
| 5 | Cardiovascular I_check list (31) | Aortic arch , Brachiocephalic a., Left common carotid a., Left subclavian a., Superior vena cava, inferior vena cava, Ascending aorta, Pulmonary trunk, right pulmonary a. , Left pulmonary a., right atrium, Left atrium, Right coronary a., left coronary a., Right marginal a., Right ventricle, left ventricle, Apex of heart, Anterior interventricular a., posterior interventricular a., Left pulmonary v., right pulmonary v., Pulmonary valve, Tricuspid valve, Bicuspid valve, Papillary muscle, Great cardiac v., middle cardiac v., small cardiac v., coronary sinus, Pectinate muscle, |
| 6 | Cardiovascular II_check list (26) | Superior vena cava, inferior vena cava, Internal carotid a., External carotid a. , right Common carotid a., right subclavian a., Abdominal aorta, Celiac trunk, Superior mesenteric a. , inferior mesenteric a., Splenic a., renal a., testicular or ovarian a., testicular or ovarian v. , brachial a., radial a., ulnar a., Renal a., Common iliac a., Internal iliac a., External iliac a., Femoral a., popliteal a., anterior tibial a., posterior tibial a., fibular a., |
| 8 | Male _check list (19) | Scrotum, Testis, Testicular a., Pampiniform plexus, Cremaster m., Epididymis, Spermatic cord, Ductus deferens, Ejaculatory duct, Prostate gland, Seminal vesicle , Bulbourethral gland, Urogenital diaphragm , Glans penis, Penis, Corpus spongiosum, Corpora cavernosa, Suspensory ligament of penis , Deep dorsal vein (of penis), |
| 9 | Female _check list (21) | Ovary, Uterine tube, Fimbriae, Infundibulum, Ampulla, Isthmus, Ovarian lig., Broad lig., Uterus, Fundus of uterus , Body of uterus, Cervix, Vagina, Fornix, Clitoris, Labia majora, Labia minora, uterus isthmus , vaginal orifice, urinary bladder, rectum, |
| 10 | Respiratory_Checklist (30): | Frontal sinus, Sphenoid sinus, Septal cartilage, Epiglottis, Vestibular fold (False vocal cord), Vocal cord (true vocal fold) , Pharyngeal tonsil , auditory tube, Superior nasal meatus, Middle nasal meatus, inferior nasal meatus, Superior nasal concha, middle nasal concha, inferior nasal concha , Thyroid cartilage, Cricoid cartilage, Arytenoid cartilage, Corniculate cartilage, Trachea, Tracheal cartilage, Primary (main) bronchus, Secondary bronchus, Oblique fissure of right lung , Oblique fissure of left lung, Horizontal fissure of lung, right superior lobe of lung , Left superior lobe of lung , Right middle lobe of lung, right inferior lobe of lung, Left inferior lobe of lung, |
| Department of Medical Laboratory Science and Biotechnology | | |
| 2 | Skeletal_Checklist (36): | Vomer , C1 atlas , Dens, Superior costal facets , Transverse costal facets , Clavicle , Coracoid process , Spine of scapula , Acromion , Subscapular fossa , Deltoid tuberosity , Trochlea, Capitulum, Olecranon fossa, Coronoid fossa, trochlear notch , Olecranon process , Coronoid process, Styloid process of ulna, Styloid process of radius, Acetabulum, Iliac crest , Iliac fossa , Ischial spine , Ischial tuberosity , Obturator foramen , Pubic symphysis , Greater trochanter , Lesser trochanter, Neck of femur, Patellar surface, Patella , Tibial tuberosity , Medial malleolus , Lateral malleolus , Head of fibula , |
| 3 | Muscular I_Checklist (22): | frontal belly of occipitofrontalis, Occipital belly of occipitofrontalis, Orbicularis oculi , Zygomaticus major, Zygomaticus minor , Risorius , Levator labii superioris, Depressor labii inferioris, Depressor anguli oris, mentalis, Orbicularis oris , Buccinator , Masseter, Temporalis , sternal head of sternocleidomastoid , clavicular head of sternocleidomastoid , Trapezius, Levator scapulae , Latissimus dorsi, Diaphragm, Rhomboid major , Rhomboid minor, |
| 4 | Muscular II_Checklist (30): | Pectoralis minor, Serratus anterior, Pectoralis major, Deltoid, External oblique abdominis , Internal oblique abdominis, Transversus abdominis, Rectus abdominis, Triceps brachii, Biceps brachii, Brachialis, Brachioradialis, Sartorius, Adductor longus, Adductor brevis, Adductor magnus, Rectus femoris of Quadriceps femoris, Vastus lateralis of Quadriceps femoris , Vastus medialis of Quadriceps femoris, Vastus intermedius of Quadriceps femoris , Gluteus maximus, Gluteus medius, biceps femoris , semitendinosus , semimembranosus, Gastrocnemius, Soleus, Flexor digitorum longus, Flexor hallucis longus, Tibialis posterior, |
| 5 | Nervous system II　Checklist (25): | Olfactory n., optic n., oculomotor n., trochlear n., trigeminal n., abducens n., facial n., vestibulocochlear n., glossopharyngeal n., vagus n., accessory n., hypoglossal n., Sympathetic trunk, Axillary nerve, Musculocutaneous nerve , Median nerve, Ulnar nerve, Radial nerve, Femoral nerve, Obturator nerve , Sciatic nerve, Tibial nerve, Common fibular nerve, superior fibular nerve, deep fibular nerve, |
| 6 | Nervous system I Checklist (25): | Transverse fissure, Longitudinal fissure , Frontal lobes, parietal lobes, temporal lobes, occipital lobes, insula, Central sulcus, Lateral sulcus, Hypothalamus, Pituitary gland, Optic chiasm, Lateral ventricles, Third ventricle, cerebral aqueduct, Fourth ventricle, Cerebral peduncles, superior colliculi, inferior colliculi, Pons, Middle cerebellar peduncles, Medulla oblongata, Olive, Cerebellum, Flocculonodular lobe, |
| 9 | Cardiovascular I_Checklist (30): | Aortic arch, Brachiocephalic a., Left common carotid a., Left subclavian a., Superior vena cava, inferior vena cava, Ascending aorta, Pulmonary trunk, right pulmonary a., Left pulmonary a., right atrium,, Left atrium , Right coronary a., left coronary a., Right marginal a., Right ventricle, left ventricle, Apex of heart, Anterior interventricular a. , posterior interventricular a. , Left pulmonary v., right pulmonary v., Pulmonary valve, Tricuspid valve, Bicuspid valve, Papillary muscle, Great cardiac v., middle cardiac v., small cardiac v., Pectinate muscle, |
| 10 | Cardiovascular II_Checklist (19) | Hepatic vein, Hepatic portal vein, Fibular a., Posterior tibial a., Anterior tibial a., Popliteal a., Internal iliac a., Gonadal a., Ulnar a., Brachial a., Femoral a., Axillary a., Thoracic aorta, Abdominal aorta, Inferior mesenteric a., Superior mesenteric a., Celiac trunk, External carotid a., Internal carotid a., |
| 11 | Respiratory Checklist (30): | Frontal sinus, Sphenoid sinus, Maxillary sinus, Septal cartilage, Vomer, Epiglottis, Vestibular fold (false vocal cord) , Vocal cord (true vocal fold), Pharyngeal tonsil, Superior nasal meatus, middle nasal meatus, inferior nasal meatus, Superior nasal concha, middle nasal concha, inferior nasal concha, Thyroid cartilage, Cricoid cartilage, Trachea , Tracheal cartilage , Lung, Primary (main) bronchus, Secondary bronchus, Oblique fissure of right lung, Oblique fissure of left lung, Horizontal fissure of lung, right superior lobe of lung, Left superior lobe of lung, Right middle lobe of lung, right inferior lobe of lung, Left inferior lobe of lung, |
